# The Index Medicus

**Published:** 1888-04

**Authors:** 


### THE INDEX MEDICUS.

It seems only natural to presume that out of the large number of physicians in America alone, a sufficient number would be found to subscribe for the *Index Medicus* to meet the current expense of publishing it. As supplementing the *Index Catalogue* of the library of the Surgeon-General's office, it is unique and worthy of liberal support, yet we are assured by the present publisher of it, Mr. George S. Davis, of Detroit, that it is not self-supporting, and the same was the case in the hands of its first publisher. It is not to be supposed that a publisher will continue, indefinitely, to publish at an annual loss to himself, even so important and so desirable a reference index as this, and we venture to express the hope that a large enough number of members of our profession, both in this and other countries, will be sufficiently interested in the continuance of a work so valuable for reference as to subscribe for it, and to make it, at least, self-supporting. If this venture should prove a failure from lack of financial support, it is probably safe to assume that another will not soon be made, and its disappearance from our medical literature would be a matter of lasting regret. The work necessary in its preparation, and the thoroughness with which that work has always been done in this Index, entitle it to generous support, which we bespeak for it.

